# Establishing legal limits for driving under the influence of marijuana

**DOI:** 10.1186/s40621-014-0026-z

**Published:** 2014-10-29

**Authors:** Kristin Wong, Joanne E Brady, Guohua Li

**Affiliations:** 1 Department of Anesthesiology, College of Physicians and Surgeons, Columbia University, 622 West 168th Street, New York, 10032 NY USA; 2 Department of Epidemiology, Mailman School of Public Health, Columbia University, 722 West 168th Street, New York, 10032 NY USA; 3 Center for Injury Epidemiology and Prevention, Columbia University Medical Center, 722 West 168th Street, Room 524, New York, 10032 NY USA

**Keywords:** Drugged driving, Marijuana, Cannabis, Per se, Legal limit, Psychomotor impairment

## Abstract

Marijuana has become the most commonly detected non-alcohol substance among drivers in the United States and Europe. Use of marijuana has been shown to impair driving performance and increase crash risk. Due to the lack of standardization in assessing marijuana-induced impairment and limitations of zero tolerance legislation, more jurisdictions are adopting per se laws by specifying a legal limit of Δ^9^-tetrahydrocannabinol (THC) at or above which drivers are prosecuted for driving under the influence of marijuana. This review examines major considerations when developing these threshold THC concentrations and specifics of legal THC limits for drivers adopted by different jurisdictions in the United States and other countries.

## 1 Introduction

Drugged driving is a safety concern of increasing importance in the United States and in Europe (Asbridge [2014]; Brady and Li [2014]; Li et al. [2013]; Whitehill et al. [2014]; Wolff and Johnston [2014]). According to the 2012 National Survey on Drug Use and Health, 10.3 million individuals aged 12 years or older operated a motor vehicle under the influence of illicit drugs in 2011 (SAMHSA [2013]). Driving under the influence of drugs has also been identified as a priority area for drug control research and interventions by the Office of National Drug Control Policy and the Department of Transportation (ONDCP [2013]). Marijuana, the most commonly detected non-alcohol substance in American and European drivers, has been shown to impair driving performance and cognitive functions and increase crash risk (Asbridge [2014]; Downey et al. [2013]; Hartman and Huestis [2013]; Li et al. [2013]; Ramaekers et al. [2004], [2006]; Schwope et al. [2012]). While driving under the influence of marijuana is prohibited in all US states (Walsh [2009]), there has been a significant rise in marijuana decriminalization and medical marijuana legalization over the past decade (NORML) [2014c]; Svrakic et al. [2012]; Wolff and Johnston [2014]). Recreational marijuana use is also currently legal in Colorado and Washington (Asbridge [2014]; Barry et al. [2014]; Kilmer [2014]). The recent increase in marijuana accessibility (Sewell et al. [2009]; Whitehill et al. [2014]) underscores the need for effective legislation to combat potential health and safety risks posed by driving under the influence of marijuana (Grotenhermen et al. [2007]; Whitehill et al. [2014]).

In collaboration with the National Highway Traffic Safety Administration (NHTSA) and the International Association of Chiefs of Police (IACP), law enforcement agencies have developed drug evaluation and classification (DEC) programs in the United States since 1989 (DuPont et al. [2012]; Romano and Voas [2011]; Talpins and Hayes [2004]). DEC programs are the principal legal strategy to enforce drugged driving legislation, and train police officers to recognize signs of drug-induced impairment in drivers [e.g., bloodshot eyes, increased nervousness, etc. (Wolff and Johnston [2014])], qualifying them as drug recognition experts (DREs) (ONDCP [2010]; Asbridge [2014]; Talpins and Hayes [2004]; Walsh [2009]). Similar programs also exist in Europe (EMCDDA [2009]; Verstraete et al. [2011]), but as of 2009, training was required for traffic police in only 4 countries (Belgium, Portugal, Sweden, and United Kingdom) (EMCDDA [2009]). If a case involving a drugged driver goes to trial and a DRE was present at the scene, the DRE may testify for the prosecution and contribute his expert opinion on the degree of the driver’s impairment DuPont et al. [2012]; Talpins and Hayes [2004]). In the United States, over 6,000 law enforcement officers have been certified as DREs (ONDCP [2010]). Although DREs have increased the number of drugged driving arrests, DRE evaluations are generally only performed when a driver’s blood alcohol concentration (BAC) results are inconsistent with the driver’s performance on Standardized Field Sobriety Tests (Talpins and Hayes [2004]). The limited use of DRE evaluations means that drugged drivers with high BACs may bypass DRE evaluations, leading to underreporting of drugged driving (EMCDDA [2009]). Furthermore, in the absence of specified legal limits for drugs in most states and countries, the value of DRE evaluation and drug testing results as evidence in court is limited. This review examines major considerations when establishing legal limits of Δ^9^-tetrahydrocannabinol (THC) for drivers and specifics of per se laws regarding driving under the influence of marijuana adopted by different jurisdictions in the United States and other countries.

## 2 Review

### 2.1 Legislative approaches

There are currently three principal approaches to the assessment and control of drugged driving in the United States. The first approach is “effect-based,” by which prosecutors must prove that the drug impaired the driver’s ability to operate a motor vehicle (Grotenhermen et al. [2007]). Although this approach is implemented in the majority of states in the United States, it is difficult and complex to enforce, particularly due to the lack of standardization in methods of assessing and determining drug-induced impairment (Kay and Logan [2011]; Romano and Pollini [2013]). A uniformly accepted definition of impairment does not exist for different drugs, and these definitions vary across states (DuPont et al. [2012]). Laboratory analysis of blood or urine samples is also expensive and time-consuming, and in general, these tests are only performed if a driver has a BAC within the legal limit (<0.08%) (DuPont et al. [2012]). As a result, in comparison to drunk driving, drugged driving is rarely prosecuted in the United States (DuPont et al. [2012]; Walsh [2009]).

Due to the limitations of the effect-based approach, some states have adopted “per se” laws, which make it a criminal offense for an individual to have a specified amount of drug or metabolite in his or her body while operating a motor vehicle (Asbridge [2014]; DuPont et al. [2012]; Grotenhermen et al. [2007]; Lacey et al. [2010]). This threshold concentration is a legal limit, and exceeding this amount serves as proof of legal impairment (Asbridge [2014]; Ramaekers et al. [2006]).

The third approach is the “zero tolerance” policy, a special form of the per se law in which the legal limit is set at zero or the minimum reliably detectable level (Asbridge [2014]; Grotenhermen et al. [2007]; Lacey et al. [2010]; Reisfield et al. [2012]; Salomonsen-Sautel et al. [2014]). However, zero tolerance laws pose several shortcomings. Such laws incriminate drivers whose bodily fluids contain any amount of drug or metabolite, and who therefore may not actually be impaired while driving (Grotenhermen et al. [2007]; Ramaekers et al. [2006]). This issue is exacerbated for marijuana use. THC has a half-life of approximately 7 days, and the total elimination of a single dose from one’s urine can take up to 30 days (Ashton [2001]; Bedard et al. [2007]). Due to its relatively long half-life, THC can be detected in the blood and urine of an individual for hours to days and for days to months following marijuana use, respectively, depending on the frequency of use and other factors (NORML [2014b]; Asbridge [2014]; Grotenhermen et al. [2007]; NORML [2014b]). For instance, chronic marijuana users have plasma THC concentrations ranging from 1.0 μg/L to 11.0 μg/L, which are maintained by the continual passage of THC from the tissues into the bloodstream (Wolff and Johnston [2014]). Therefore, habitual users who test positive for THC may not necessarily be impaired at the time of testing (Bedard et al. [2007]). Another major limitation of zero tolerance laws is the risk to convict those with heavy passive exposure to marijuana smoke (Grotenhermen et al. [2007]; Wolff and Johnston [2014]). Following passive exposure, the THC of an individual may be detectable in his blood in concentrations <1.0 μg/L within one hour, without inducing concurrent impairment (Grotenhermen et al. [2007]; Sharma et al. [2012]; Wolff and Johnston [2014]).

European countries also use different legal approaches in tackling drugged driving (EMCDDA [2009]; Verstraete et al. [2011]). The impairment approach, which is comparable to the effect-based law in the United States, is enforced in 11 European countries (Gjerde et al. [2011a]; Verstraete et al. [2011]; Wolff and Johnston [2014]). Like some US states, five European countries employ per se laws, which prosecute a driver if his body fluid surpasses a defined cut-off concentration (Gjerde et al. [2011a]; Vindenes et al. [2012]; Wolff and Johnston [2014]). As in the US, zero tolerance laws in European countries also fall under the per se category (Verstraete et al. [2011]; Wolff and Johnston [2014]). Nine European countries have adopted the “two-tier system,” a combination of the impairment-based law and the per se law. The two-tier system penalizes drivers with any amount of an illicit drug in his body with a non-criminal or lower-level criminal fine, and more severely penalizes drivers who are impaired by any substance (EMCDDA [2009]; Verstraete et al. [2011]).

### 2.2 Considerations when developing legal THC limits for driving

#### 2.2.1 Method of marijuana consumption

The rate of marijuana absorption into the bloodstream and body tissues is determined by the route of drug administration (Huestis [2007]). Inhalation and ingestion of marijuana produce psychoactive effects of different duration and intensities (Grotenhermen et al. [2007]; Hall and Degenhardt [2009]; Schwope et al. [2012]; Wolff and Johnston [2014]). When smoking marijuana is the principal method of consumption, there is a fast release of THC via inhalation into the general circulation (Huestis [2007]). Subsequent absorption of inhaled THC is also rapid (Wolff and Johnston [2014]), and THC can be detected within seconds after the initial puff of a cigarette (Schwope et al. [2012]). Blood concentrations typically reach a maximum within 3 to 15 minutes of intake (Verstraete [2004]; Wolff and Johnston [2014]). Furthermore, the quantity of marijuana in an average rolled cigarette is about 200 mg, which is equivalent to approximately 5 mg to 30 mg active THC (Wolff and Johnston [2014]). On the other hand, absorption of orally ingested forms of THC occurs slowly and unpredictably due to its high octanol/water partition coefficient (Huestis [2007]) and its passage through the gut (Wolff and Johnston [2014]). Maximal blood concentrations are lower for ingested marijuana (Huestis [2007]), and are reached between 1 and 7 hours after consumption (Huestis [2007]; Wolff and Johnston [2014]). Edibles (ingested forms of marijuana) also contain varied amounts of THC (e.g., brownies, pumpkin cake, chocolate cookie), ranging from 20 mg to 64 mg (Huestis [2007]; Wolff and Johnston [2014]).

#### 2.2.2 Frequency of marijuana use

Occasional and recreational users have lower plasma THC concentrations than regular and frequent users (Downey et al. [2013]; Wolff and Johnston [2014]). However, the effect of THC consumption on impairment of driving performance may be higher for occasional marijuana users than for frequent users (Bosker et al. [2012]; Downey et al. [2013]). A meta-analysis of more than 120 studies demonstrated that regular users were less impaired than occasional users at the same THC concentration (Wolff and Johnston [2014]), which may be attributed to physiological tolerance or learned compensatory driving behavior (DuPont et al. [2012]; Grotenhermen et al. [2007]; Wolff and Johnston [2014]). Habitual users are more susceptible to experiencing chronic, long-term effects of marijuana, including withdrawal symptoms and developing addiction to the drug (Ashton [2001]; Hall and Degenhardt [2009]; Karila et al. [2014]; Lee et al. [2014]). On the other hand, infrequent users are more prone to experiencing stronger acute effects, like impairment (Hall and Degenhardt [2009]; Karila et al. [2014]; Moskowitz [1985]; Wolff and Johnston [2014]).

#### 2.2.3 Concurrent use of marijuana and alcohol

According to toxicological testing data, approximately 25% of drivers injured in motor vehicle crashes test positive for 2 or more drugs, with marijuana and alcohol being the most common combination (Brady and Li [2013]; Kay and Logan [2011]; Li et al. [2013]). Wilson et al. ([2014]) found that 54.9% of marijuana-positive drivers in the United States had elevated BACs. Various studies have demonstrated that the combined use of marijuana and alcohol is associated with significantly greater cognitive impairment and crash risk than the use of one alone (Asbridge [2014]; Downey et al. [2013]; Ramaekers et al. [2004]; Sewell et al. [2009]).

#### 2.2.4 Polydrug use

Another major factor to be considered when developing per se laws is polydrug use, or the use of 2 or more non-alcohol drugs (Brady and Li [2013]; Hartman and Huestis [2013]; Li et al. [2013]; Romano and Voas [2011]). In Europe, 20-30% of marijuana use among drivers was combined with other psychoactive substances in 2012, with marijuana being the most frequently detected drug used simultaneously with cocaine and benzodiazepines (Wolff and Johnston [2014]). Li et al. ([2013]) found that polydrug use was associated with a 3.41-fold increased risk of involvement in a fatal crash. Legislation for multiple drug use is complicated, for limited drug-drug combinations have been fully characterized in the context of drugged driving, and possible interactions range widely (Wolff and Johnston [2014]). This makes it difficult for police officers to recognize impairment induced by several drugs and to conduct simultaneous roadside screenings for multiple substances (Wolff and Johnston [2014]).

#### 2.2.5 Testing method and specimen collection

In order to measure the level of THC present in a driver, states conduct chemical screening tests via collection of the driver’s blood, urine, and/or oral fluid (OF) (NORML [2014a]; Walsh [2009]). Blood sampling is the most effective method, but it is also the most invasive (Wolff and Johnston [2014]). There is a lack of standardization regarding blood sample collection (i.e., duration of time after a driver is pulled over is his blood to be collected) and transportation conditions to a collection facility (e.g., hospital) as well. In addition, blood screening can also detect metabolites of THC [e.g., carboxy-THC (THC-COOH)] (NORML [2014b]). However, detection of THC-COOH only (no THC) does not indicate impairment (NORML [2014b]; Sewell et al. [2009]). Furthermore, active 11-hydroxy-THC, a metabolite that continues to produce effects in the body after being metabolized, is detected in very low concentrations in blood after smoking marijuana (Himes et al. [2013]). Therefore, there is little cross-reaction from active 11-hydroxy-THC and inactive metabolites with THC-COOH antibodies, which would typically cause extension of a screening test’s detection window (Wolff and Johnston [2014]).

The majority of states in the United States also conduct urine screening tests, or urinalyses (NORML [2014a]; Walsh [2009]). Urine screening tests are generally performed via immunoassays that can detect combinations of THC and its metabolites, but not necessarily THC alone (Sewell et al. [2009]; Wolff and Johnston [2014]). Therefore, urinalysis is only reflective of previous marijuana exposure, rather than degree of impairment by the drug (Huestis [2007]; Sewell et al. [2009]; Wolff and Johnston [2014]).

Recently, there has been strong interest in collecting bodily fluids other than blood and urine in order to facilitate roadside drug testing (Himes et al. [2013]; Huestis [2007]). The use of OF to determine a driver’s THC level has gained popularity in the United States, for it is the easiest and least invasive method (Himes et al. [2013]; Wolff and Johnston [2014]). Australia currently collects OF samples for its random roadside marijuana testing programs (Asbridge [2014]; Chu et al. [2012]; Davey and Freeman [2009]; Wolff and Johnston [2014]). Samyn et al. ([1999]) demonstrated that OF reflects whether an individual has a drug present in his blood better than urine (Gjerde et al. [2011b]). Although the THC concentration in OF correlates strongly with that in blood on the population level, it remains problematic to estimate blood THC concentrations based on OF samples for individual drivers (Gjerde et al. [2011b]; Ramaekers et al. [2006]). In addition, OF sample volumes are significantly lower than those for urine, potentially resulting in insufficient specimen for confirmation testing (Huestis et al. [2011]). Numerous studies have investigated whether OF THC concentration is an accurate measure of blood THC concentration as an indicator of driving impairment (Gjerde et al. [2011b]; Wolff and Johnston [2014]). A study conducted by Ramaekers et al. ([2006]) found that there was a linear relationship between magnitude of performance impairment and THC level in OF, and that OF containing THC may be indicative of recent exposure to or consumption of marijuana.

#### 2.2.6 Pharmacokinetics

THC is extremely lipid soluble (Ashton [2001]; Huestis [2007]; Nahas [2001]; Wolff and Johnston [2014]), and is widely distributed in the body to tissues at rates dependent on blood flow (Ashton [2001]). THC in blood rapidly decreases over time (Huestis [2007]), typically declining to 1.0-4.0 μg/L within 3–4 hours (Wolff and Johnston [2014]). Therefore, delayed blood collection often results in an inaccurate THC measurement, which may not be reflective of a driver’s level of impairment while driving. Depending on the frequency of marijuana use, THC in blood can be detectable for hours to days following the most recent use (NORML [2014b]; Verstraete [2004]). In addition, THC and THC-COOH are predominantly detected in blood plasma, where 95-99% of the metabolites are bound to lipoproteins (Huestis [2007]). THC concentrations in whole blood are approximately one-half the concentrations in plasma (Barceloux [2012]; Huestis [2007]).

Marijuana as detected by urinalysis has a long-ranging detection window in regular and non-regular users, primarily due to metabolic cross-reaction between active 11-hydroxy-THC and inactive metabolites (Wolff and Johnston [2014]). Detection times in urine depend on pharmacological factors, including method of THC consumption and the metabolism rate of an individual (Huestis [2007]), and metabolites are typically not detectable in urine for up to 4 hours (Wolff and Johnston [2014]). Metabolites can be detected in urine for several days or months, contingent upon the frequency of use (NORML [2014b]; Ramaekers et al. [2006]; Verstraete [2004]). Due to the long detection window of THC in urine (Wolff and Johnston [2014]), a positive immunoassay test may only be indicative of previous use (Huestis [2007]; Ramaekers et al. [2004]; Wolff and Johnston [2014]). The long excretion half-life of THC-COOH, particularly in chronic marijuana users, makes it difficult to determine the timing of past drug use (Huestis [2007]).

There is little to no THC-COOH metabolite present in OF (Huestis [2007]), meaning the detection window for OF is not overextended due to any metabolite cross-reaction (Himes et al. [2013]; Wolff and Johnston [2014]). Since detection times of cannabinoids in OF are shorter (i.e., hours to days after use) (Verstraete [2004]; Wolff and Johnston [2014]), OF is more reflective of recent cannabis use than urine (Huestis [2007]). Furthermore, during the smoking of marijuana, the oral mucosa is exposed to high THC concentrations, serving as the main source for release of THC into OF (Huestis [2007]; Huestis and Cone [2004]). Peak OF THC concentrations of 5800 μg/L have been reported immediately after smoking marijuana, followed by 30 minutes of rapid elimination and a subsequent slower decrease (Huestis and Cone [2004]; Wolff and Johnston [2014]).

#### 2.2.7 Legal THC limits for driving adopted by different jurisdictions

In the United States, there are currently seven states that enforce legal marijuana metabolite limits for drivers (Colorado, Iowa, Montana, Nevada, Ohio, Pennsylvania, and Washington) (Table 1). With the exception of Iowa, all of these states list legal THC thresholds in whole blood in their respective drugged driving laws. Iowa, Nevada, Ohio, and Pennsylvania have set legal THC and/or THC-COOH limits in urine. It is important to note that the drugged driving legislation for Iowa specifies a legal limit for THC-COOH, not for THC, most likely because urine screening tests cannot detect THC alone. Despite this limitation of urinalysis, Nevada, Ohio, and Pennsylvania specified legal concentrations of THC in urine. In addition, these states have legal limits for both THC as well as its metabolites (THC-COOH). Ohio also enforces a legal limit for THC-COOH “in combination with alcohol or other drugs” to account for the heightened impairment caused by the use of marijuana with other drugs. Furthermore, all seven states collect at least one specimen for marijuana testing, but respective limits are not indicated for all collected specimens (Table 1). For instance, in Colorado, a driver’s blood, urine, and OF may be collected, but state legislation only denotes a legal THC limit in blood. Drug screenings based on an unspecified specimen provide preliminary positive/negative tests, which reflect whether an individual has any amount of drug in his body regardless of potential impairment. A positive screening test gives a police officer reasonable cause to ask the driver to provide a blood sample to quantify his THC level. If a driver refuses to participate in this initial screening process, his test is considered positive.

**Table 1:** **Legal Δ**^**9**^-**tetrahydrocannabinol** (**THC**) **thresholds for drivers in states with per se laws**

State	Legal THC limit	Collected specimen	Year effective
Colorado	5.0 μg/L in blood	Blood, urine, or OF	2013
Iowa	THC-COOH: 50.0 μg/L in urine	Blood or urine	2010
Montana	5.0 μg/L in blood	Blood	2013
Nevada	THC: 10.0 μg/L in urine, 2.0 μg/L in blood THC-COOH: 15.0 μg/L in urine, 5.0 μg/L in blood	Blood, urine, or other bodily substance	2003
Ohio	THC: 10.0 μg/L in urine, 2.0 μg/L in blood THC-COOH: 35.0 μg/L in urine, 50.0 μg/L in blood THC-COOH in combination with alcohol or other drugs: 15.0 μg/L in urine, 5.0 μg/L in blood	Blood, urine, or other bodily substance	2006
Pennsylvania	THC or THC-COOH: 1.0 μg/L in blood or urine	Blood or urine	2011
Washington	5.0 μg/L in blood	Blood	2013

*Abbreviations*: *OF* oral fluid, *THC* Δ^9^-tetrahydrocannabinol, *THC*-*COOH* carboxy-Δ^9^-tetrahydrocannabinol.

*Sources*: (NORML). Drugged Driving. Washington, D.C. 2014a. http://norml.org/legal/drugged-driving. Accessed July 15 2014.; Lacey J, Brainard K, Snitow S. Drug Per Se Laws: A Review of Their Use in States. Washington, D.C.: National Highway Traffic Safety Administration (NHTSA). 2010. Accessed July 15 2014.; Avila EN. Minimum Levels of Controlled Substances or Their Metabolites in Blood to Establish Presence of Controlled Substance. *Pennsylvania Bulletin*. [2011] April 30. Accessed July 23 2014.; [Wash Rev Code § 46.61.502]); Walsh JM. A State-by-State Analysis of Laws Dealing With Driving Under the Influence of Drugs. Washington, D.C.: National Highway Traffic Safety Administration (NHTSA). 2009. Accessed July 12 2014.

Sixteen countries in Europe have set legal non-zero THC concentrations at or above which drivers are prosecuted for driving under marijuana (Table 2). Of these countries, eleven enforce legal limits in whole blood (Denmark, Finland, France, Greece, Ireland, Italy, Norway, Poland, Portugal, Switzerland, and United Kingdom), and four enforce legal limits in blood serum (Belgium, Germany, Luxembourg, and Slovenia). Netherlands has implemented legal limits in both blood and blood serum. Unlike the United States, no European countries have set legal THC limits in urine. Furthermore, several European countries enforce legal thresholds for both THC and its metabolite THC-COOH (Table 2).

**Table 2:** **Types of drugged driving legislation and legal Δ**^**9**^-**tetrahydrocannabinol** (**THC**) **thresholds for drivers in European countries**

Country	Legislation	Legal THC limit
Belgium	Two-tier	1.0 μg/L in blood serum
Denmark	Two-tier	1.0 μg/L in blood
Finland	Two-tier	THC: 1.0 μg/L in blood THC-COOH: 5.0 μg/L in blood
France	Two-tier	1.0 μg/L in blood
Germany	Two-tier	1.0 μg/L in blood serum
Greece	Impairment	1.0 μg/L in blood
Ireland	Impairment	THC: 2.0 μg/L in blood THC-COOH: 5.0 μg/L in blood
Italy	Per se	THC or THC-COOH: 0.5 μg/L in blood
Luxembourg	Impairment	2.0 μg/L in blood serum
Netherlands	*	3.0 μg/L in blood** 5.0 μg/L in blood serum
Norway	Impairment	1.3 μg/L in blood***
Poland	Per se	THC: 2.0 μg/L in blood THC-COOH: 50.0 μg/L in blood
Portugal	Per se	THC: 3.0 μg/L in blood THC-COOH: 5.0 μg/L in blood
Slovenia	Per se	THC: 0.3 μg/L in blood serum THC-COOH: 5.0 μg/L in blood serum
Switzerland	Per se	1.5 μg/L in blood
United Kingdom****	Impairment	THC: 2.0 μg/L in blood THC-COOH: 10.0 μg/L in blood

*Impairment as of 2011, but a proposal for a two-tier system has been implemented.

**Recommended by Netherlands Advisory Committee.

***Norway specified THC levels comparable to BAC (with regard to severity of impairment): 3.0 μg/L in blood is comparable to 0.05% BAC; 9.0 μg/L in blood is comparable to 0.12% BAC.

****Initial drug screening is conducted via Drugalyser oral swab tests. A positive test could lead to a blood sample test to quantify THC level.

*Abbreviations*: *THC* Δ^9^-tetrahydrocannabinol, *THC*-*COOH* carboxy-Δ^9^-tetrahydrocannabinol.

*Sources*: Verstraete A, Knoche A, Jantos R, Skopp G, Gjerde H, Vindenes V et al. Driving under the Influence of Drugs, Alcohol and Medicines (DRUID): Per se limits - Methods of defining cut-off values for zero tolerance. 2011. Accessed July 20 2014; Wolff K, Johnston A. Cannabis use: a perspective in relation to the proposed UK drug-driving legislation. *Drug Test Anal*. 2014; 6(1–2):143–54.

#### 2.2.8 Recommendations for future research

To further understand the safety risk posed by marijuana use, more studies investigating the effects of marijuana on impaired driving should be conducted. Further research is also needed to provide the scientific basis for developing legal THC limits for population groups other than drivers, such as those working in safety-sensitive positions.

In addition, it is necessary to standardize testing protocols for each collected specimen (i.e., blood, urine, and OF). This would resolve the current variation in duration of time after a driver is pulled over or involved in a motor vehicle crash that his body fluid is collected. By limiting the decline of THC in the body over time, standardization of testing conditions would enable police officers to more accurately determine a driver’s degree of impairment while driving.

The rise in technology has led to the proposal of novel drug testing methods. Some studies have examined the possible detection of THC in breath, a noninvasive and easily observed drug screening method (Beck [2014]; Himes et al. [2013]). Himes et al. ([2013]) found that chronic and occasional smokers who smoked a single marijuana cigarette experienced significant decreases in THC breath concentration after controlled smoking. The window for detecting cannabinoids in breath ranged from 0.5 to 2 hours and coincided with impairment. THC-COOH was also undetected in both groups of marijuana smokers, indicating that legal marijuana metabolite limits should reflect concentrations of THC, not THC-COOH, when a breath test is conducted. The short detection window for THC indicates that breath may be a viable alternative to OF for detecting marijuana use, but only when a driver is tested <2 hours following smoking. There is also a lack of knowledge regarding the effect of passive marijuana exposure on breath THC concentrations (Himes et al. [2013]). Further research is needed to investigate the effectiveness of breath testing to assess a driver’s degree of impairment. There is also some interest in the use of sweat as an alternative biological matrix for detecting the recent use of marijuana (De Giovanni and Fucci [2013]; de la Torre and Pichini [2004]; Huestis et al. [2008]). Sweat testing is non-invasive (Huestis et al. [2008]) and is typically collected via dermal patches or wipes (De Giovanni and Fucci [2013]; de la Torre and Pichini [2004]; Huestis et al. [2008]), which enable illicit drug use to be monitored for longer detection windows than those for urine testing (de la Torre and Pichini [2004]). However, research examining the presence of THC excretion in sweat is limited (de la Torre and Pichini [2004]; Huestis et al. [2008]). Some studies have investigated the effectiveness of dermal wipes to collect the sweat of drugged drivers. While THC was detected, 11-hydroxy-THC and THC-COOH were not (Huestis et al. [2008]). Sweat testing is further limited by difficulties in sample recovery (de la Torre and Pichini [2004]). In addition, the amount of THC in sweat is significantly low, and therefore requires sensitive analytical methods (De Giovanni and Fucci [2013]; de la Torre and Pichini [2004]).

## 3 Conclusions

There is compelling evidence that marijuana use impairs cognitive functions and driving skills and increases crash risk. Due to the limitations of impairment-based and zero tolerance laws, governments are increasingly adopting per se limits at which a driver is legally defined as being impaired to safely operate a motor vehicle. Some countries in Europe have also adopted a two-tier system, which incorporates both impairment-based and per se approaches. Several factors should be considered for the establishment of per se laws, including a driver’s mode of marijuana consumption, frequency of use, and the concurrent use of marijuana with other drugs. In order to assess a driver’s THC level, police officers administer drug screenings via collection of blood, urine, and/or OF samples. It is imperative to consider the pharmacokinetics of THC, including its rapid decline in blood over time, to facilitate standardization of drug testing. Future work should include determining the dose–response relationship between THC levels and crash risk, establishing legal THC limits for other population groups, especially those with safety-sensitive occupations, and developing innovative marijuana testing methods, such as those using OF and breath samples.

## Abbreviations

BAC: blood alcohol concentration
DEC: drug evaluation and classification
DRE: drug recognition expert
IACP: International Association of Chiefs of Police
NHTSA: National Highway Traffic Safety Administration
OF: oral fluid
THC: Δ^9^-tetrahydrocannabinol
THC-COOH: carboxy-Δ^9^-tetrahydrocannabinol
